# Supplementary material for: Isotope-encoded spatial biology identifies plaque-age-dependent maturation and synaptic loss in an Alzheimer’s disease mouse model
Source: Nat Commun. 2025 Sep 1;16:8170. doi: 10.1038/s41467-025-63328-y (PMC12402145; doi:10.1038/s41467-025-63328-y)
Supplement: Supplementary file 1 — Supplementary Information [file 41467_2025_63328_MOESM1_ESM.pdf]

SUPPLEMENTARY INFORMATION

Isotope-Encoded Spatial Biology Identifies Plaque-Age-Dependent Maturation and Synaptic Loss in an Alzheimer's Disease Mouse Model

Jack I. Wood[1,2,#], Maciej Dulewicz[1,#], Alicja Szadziewska[1], Sophia Weiner[1,2,3], Junyue Ge[1], Katie Stringer[1,4], Sneha Desai[1,4], Lydia Fenson[1], Diana Piotrowska[1], Gunnar Brinkmalm[1,5], Srinivas Koutarapu[1], Haady B. Hajar[4], Kaj Blennow[1,5,6,7], Henrik Zetterberg[1,2,5,8,9,10], Damian M. Cummings[4], Jeffrey N. Savas[11], Frances A. Edwards[4] and Jörg Hanrieder[1,2,3,12*]

Affiliations
1 Department of Psychiatry and Neurochemistry, Sahlgrenska Academy at the University of Gothenburg, Mölndal Hospital, House V, S-431 80 Mölndal, Sweden
2 Department of Neurodegenerative Disease, Queen Square Institute of Neurology, University College London, Queens Square, WC1N 3BG London, United Kingdom
3 Dementia Research Centre, Queen Square Institute of Neurology, University College London, Queens Square, WC1N 3BG London, United Kingdom
4 Department of Neuroscience, Physiology and Pharmacology, University College London, Gower Street, London, United Kingdom
5 Clinical Neurochemistry Laboratory Memory Clinic, Sahlgrenska University Hospital, Mölndal Hospital, House V, S-431 80 Mölndal, Sweden
6 Paris Brain Institute, ICM, Pitié-Salpêtrière Hospital, Sorbonne University, Paris, France
7 Neurodegenerative Disorder Research Center, Division of Life Sciences and Medicine, and Department of Neurology, Institute on Aging and Brain Disorders, University of Science and Technology of China and First Affiliated Hospital of USTC, Hefei, P.R. China
8 UK Dementia Research Institute at UCL, London, UK
9 Hong Kong Center for Neurodegenerative Diseases, Clear Water Bay, Hong Kong, PR China
10 Wisconsin Alzheimer's Disease Research Center, University of Wisconsin School of Medicine and Public Health, University of Wisconsin-Madison, Madison, WI, USA
11 Department of Neurology, Feinberg School of Medicine, Northwestern University, Chicago, IL 60611, USA
12 Department of Neuropsychiatry, Sahlgrenska University Hospital, Gothenburg, Sweden

# These authors contributed equally

*Correspondence: Prof Jörg Hanrieder,

E-mail: jh@gu.se

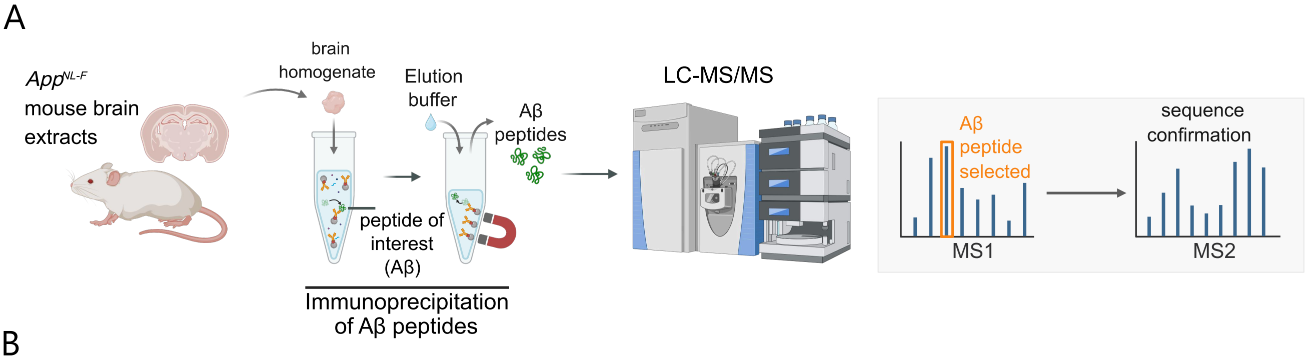

B

Gene name	Peptide sequence	Mass	Length	ppm	m/z	z	RT	PTM
APP	DAEFRHDSGYEVHHQKLVFFAEDVGSNKGAIIGLMVGG	4129,012	1-38	3,1	826,81213	5	43,179	/
APP	DAEFRHDSGYEVHHQKLVFFAEDVGSNKGAIIGLMVGGV	4228,08	1-39	2,5	846,62537	5	44,834	/
APP	DAEFRHDSGYEVHHQKLVFFAEDVGSNKGAIIGLMVGGVVIA	4511,27	1-42	1,1	903,26215	5	68,445	/

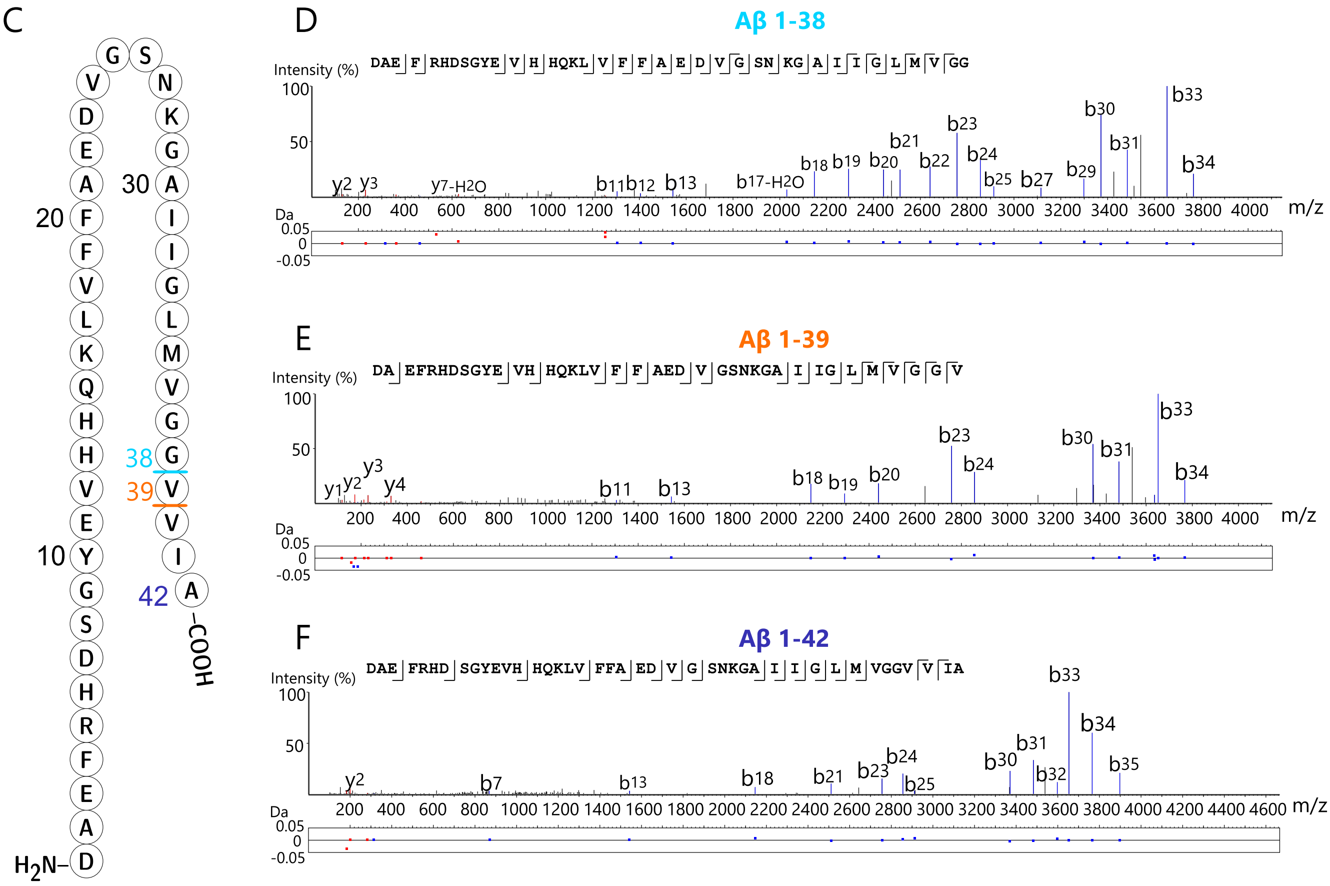

Supplementary Figure 1.

LC-MS/MS proteomic analysis confirms the presence of PTM-free Aβ1-42 in App^{NL-F} mouse brain extracts.

(**A**) Schematic overview of the anti-Aβ immunoprecipitation (IP) combined with LC-MS/MS analysis. Brain extracts from App^{NL-F} mice were homogenized, followed by immunoprecipitation of Aβ peptides. The obtained peptides were then analyzed by high-resolution LC-MS/MS to identify peptide species and potential post-translational modifications (PTMs). (**B**) Representative results from our de novo sequencing approach showing that unmodified Aβ1-42, Aβ1-39, and Aβ1-38 are the predominant peptide species in the immunoprecipitated brain extracts. (**C**) Schematic representation of the amino acid sequences of Aβ1-42, Aβ1-39, and Aβ1-38. Created with Protter. (**D–F**) Representative MS/MS spectra of Aβ1-38 (D), Aβ1-39 (E), and Aβ1-42 (F) as detected in the IP-LC-MS/MS analysis. Diagnostic b-ions confirm the presence of each peptide without PTMs. The b-ion series (charge retained on N-terminal fragment upon peptide fragmentation) dominates in several Aβ isoforms because the N-terminal region contains multiple charge carriers (e.g., Arg5, Lys16). In contrast, the C-terminal region of Aβ peptides largely lacks such charge carriers, hence, y-ions are less abundant, and fragment spectra are dominated by the b-ions.

Parts of the figure created in BioRender. Szadziewska, A. (2025) https://BioRender.com/4qpojxz

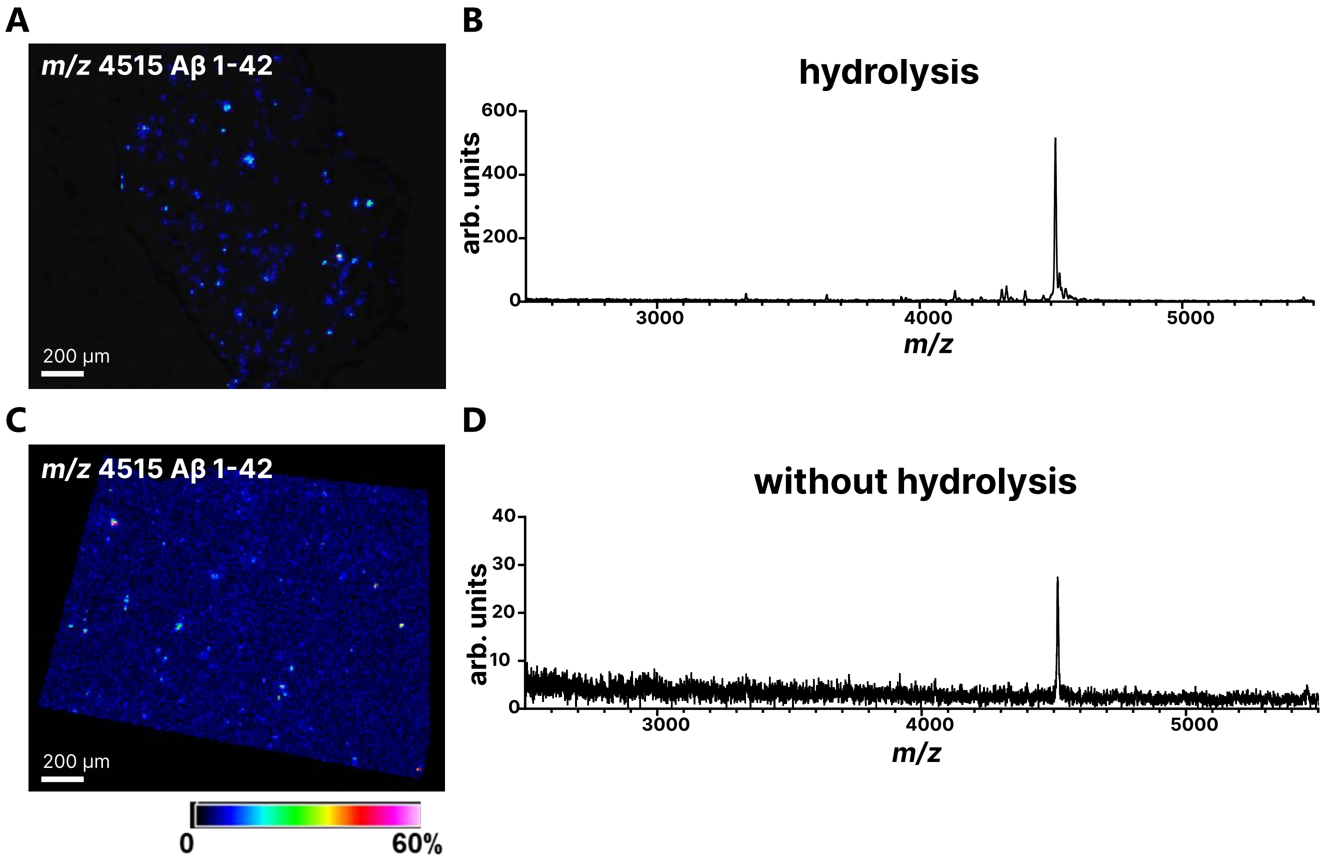

Supplementary Figure 2.

Effect of sample hydrolysis on amyloid MALDI MSI.

(**A-B**) MALDI imaging results for a tissue section prepared using formic acid hydrolysis. Corresponding single ion image and plaque ROI spectra show high intensity of Aβ signals. (**C-D**) MALDI imaging results for a tissue section prepared without formic acid hydrolysis. Detected Aβ species in plaque ROI spectra shows much lower intensity in this condition. Scale bar: 200 µm.

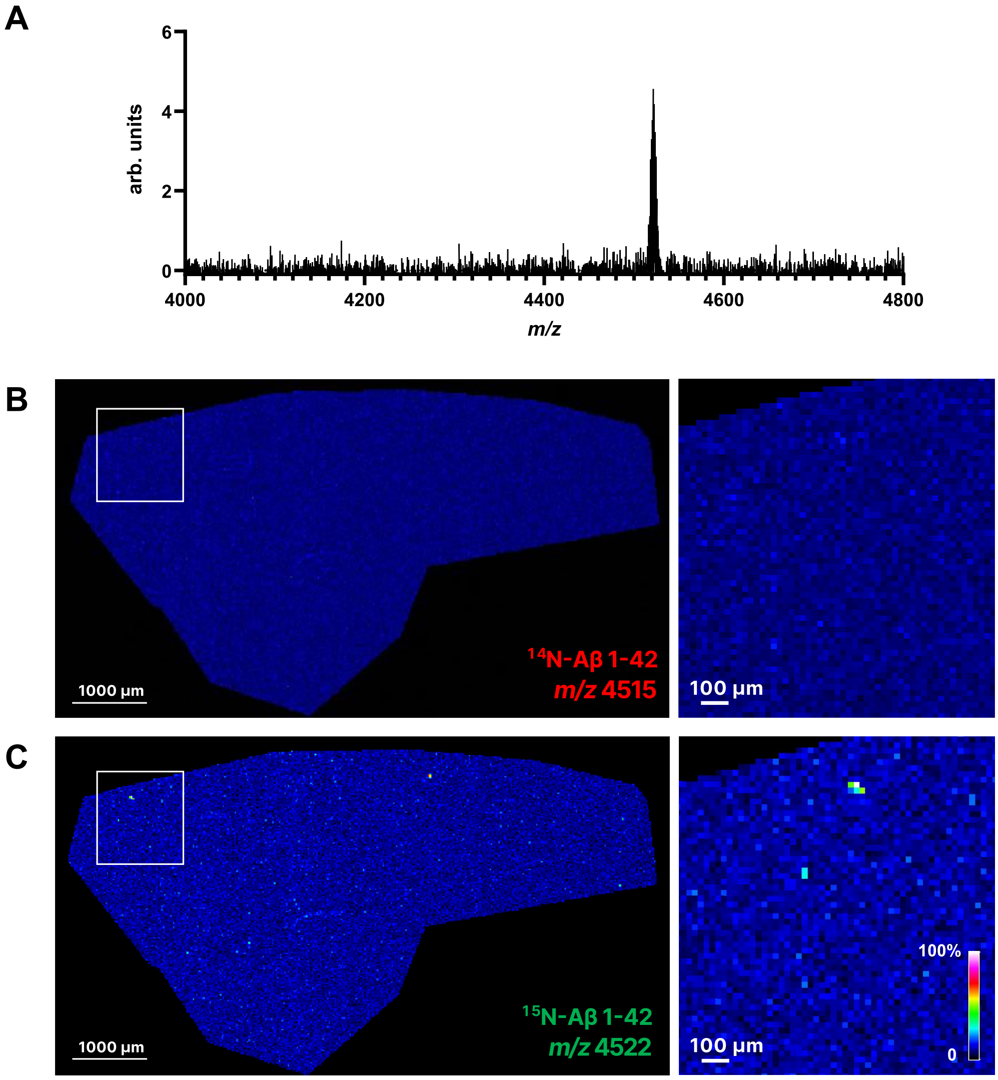

Supplementary Figure 3.

MALDI MSI analysis revealed that plaques in 10-month App^{NL-F} mice contained only ^{15}N-labelled Aβ 1-42, with no unlabelled Aβ 1-42 detected.

(**A**) The spectrum from MALDI MSI showed that the plaques in 10-month-old AppNL-F mice contained solely Aβ 1-42. (**B, C**) Single ion images of Aβ 1-42 (B) showing no unlabelled (14N) Aβ 1-42 (C) only 15N-labelled Aβ 1-42 was detected.

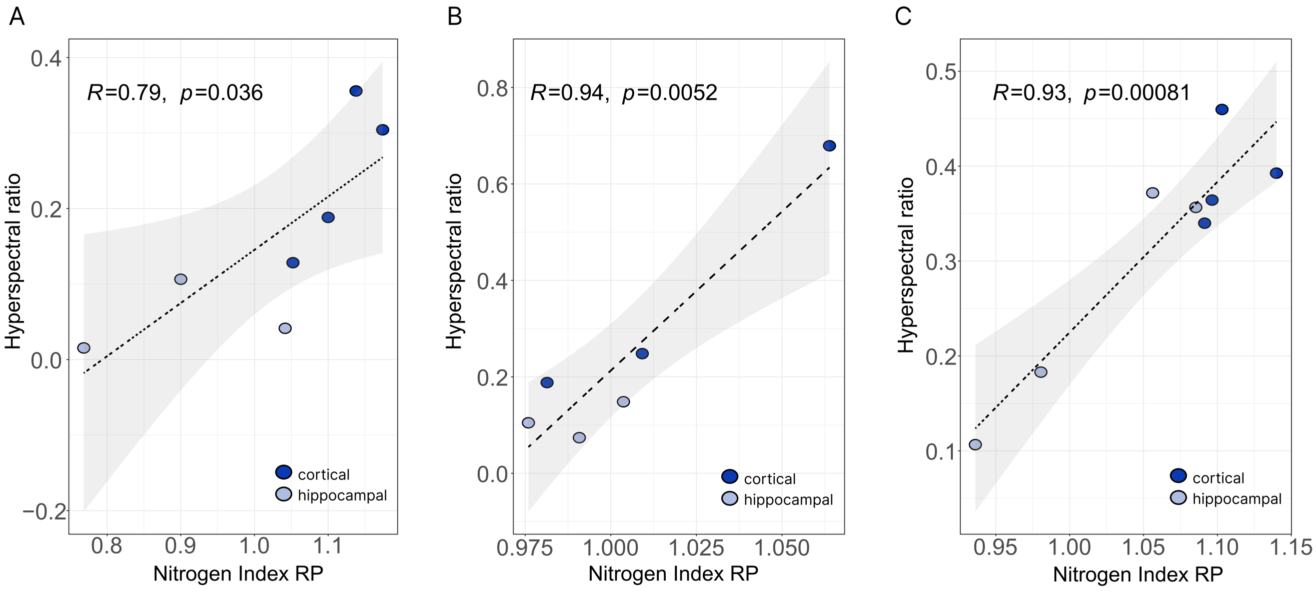

Supplementary Figure 4.

Correlation of nitrogen index in RP mode, used as a proxy for plaque age, and the LCO hyperspectral ratio in individual 18-month-old mice.

Separate correlation analyses (Pearson, two tailed) were conducted for plaques from mouse no. 6 (**A**), 4 (**B**), and 5 (**C**) (see Supplementary Table S4 for detailed information on each mouse). The results suggest a biologically robust association between the LCO hyperspectral ratio and MALDI MSI, with cortical plaques (dark blue) generally being older than hippocampal plaques (light blue).

LCO, luminescent conjugated oligothiophene; MALDI MSI, matrix-assisted laser desorption ionization mass spectrometry imaging; RP, Reflector Positive.

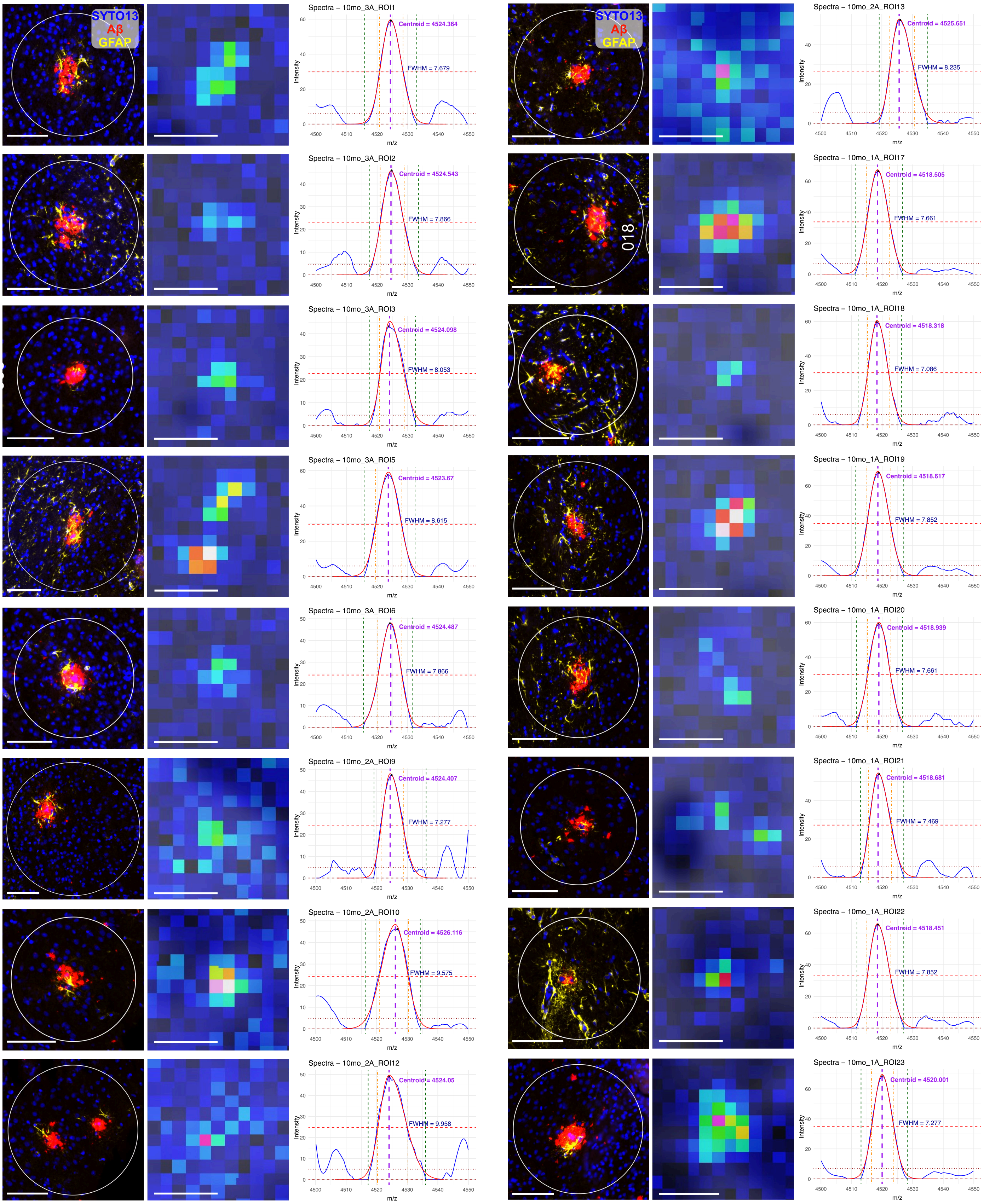

Supplementary Figure 5.

Matching Aβ signals of single plaques in 10-month-old App^{NL-F} mice (Experimental design 1) matching Aβ signals of single plaques across MALDI MSI and IHC images, including m/z spectra with peak and centroid calculations.

Scale bar: 50 µm.

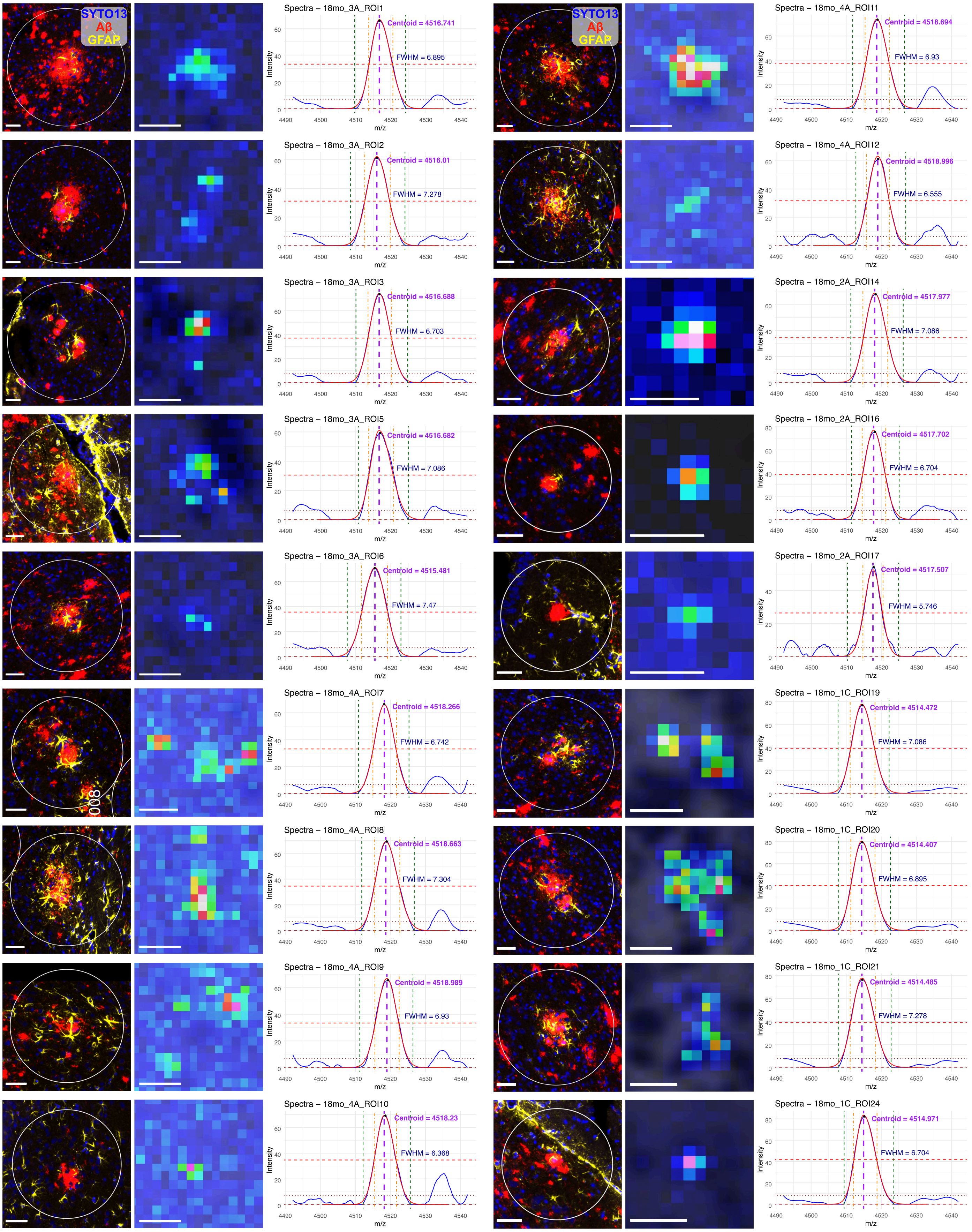

Supplementary Figure 6.

Matching Aβ signals of single plaques in 18-month-old App^{NL-F} mice (Experimental design 2) matching Aβ signals of single plaques across MALDI MSI and IHC images, including m/z spectra with peak and centroid calculations.

Scale bar: 50 μm.

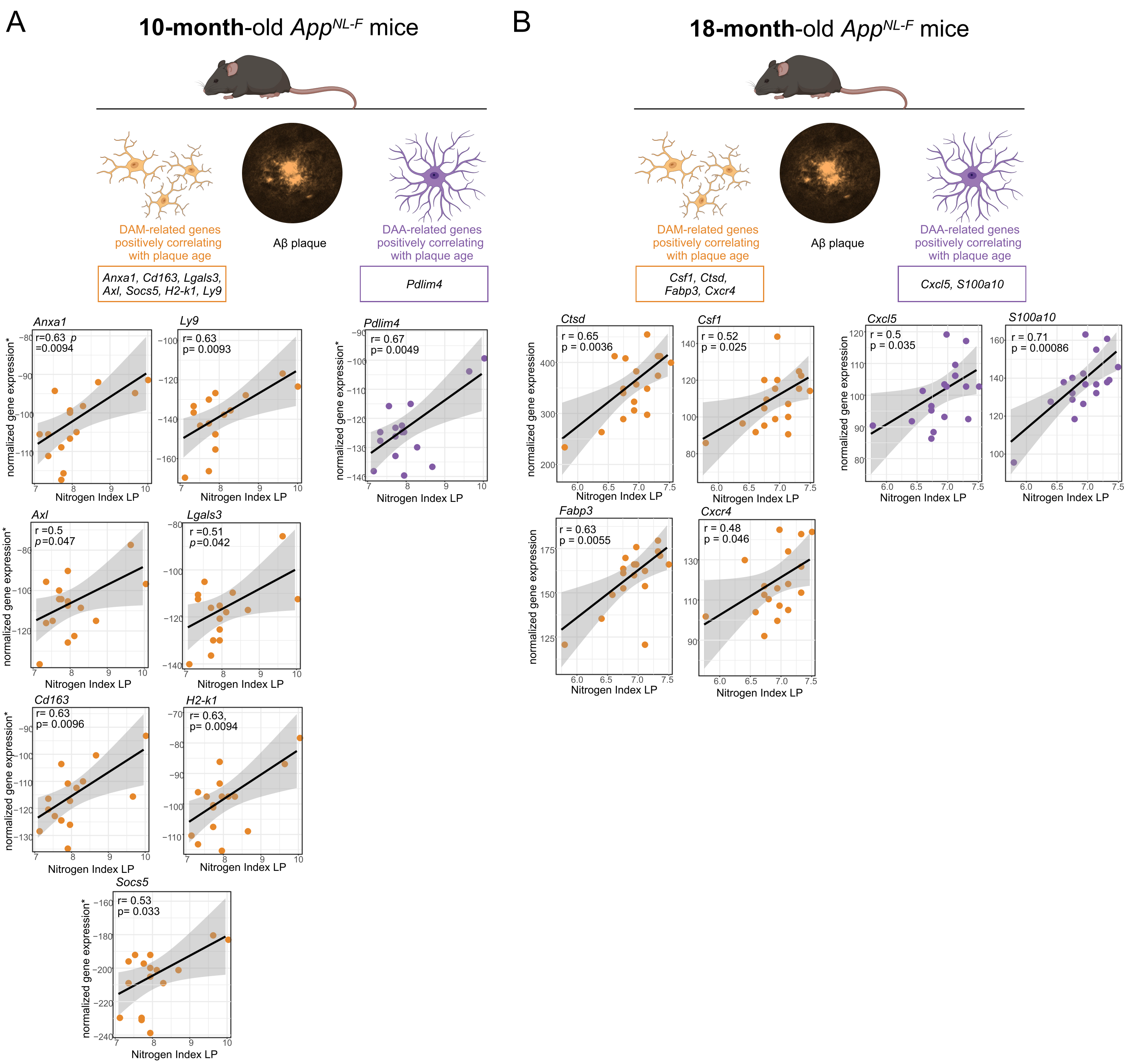

Supplementary Figure 7.

Non-exhaustive list of genes associated with disease-associated microglia (DAM) and disease associated astrocytes (DAA) showing a significant positive correlation (p>0.05) with plaque age in 10-month-old (A) and 18-month-old (B) *App*$^{NL-F}$ mice.

(A) In 10-month-old mice, the DAM-associated genes *Anxa1* (Ries et al., 2016), *Ly9* (Fitz et al., 2021), *Axl* (Keren-Shaul et al, 2017), *Lgals3* (Boza-Serrano et al, 2019), *Cd163* (Nguyen et al, 2020), *H2-k1* (Safaiyan et al., 2021), and *Socs5* (Walker et al., 2015) as well as the DAA-related gene *Pdlim4* (Habib et al., 2020) correlated significantly with plaque age (measured by the nitrogen index LP). (B) In 18-month-old mice, the DAM-associated genes *Csf1* (Keren-Shaul et al, 2017), *Ctsd* (Keren-Shaul et al, 2017), *Fabp3* (Pesämaa at al, 2023), *Cxcr4* (Bezzi et al, 2001) as well as the DAA-related gene *Cxcl5* (Habib et al., 2020) and *S100a10* (Gao et a., 2022) correlated significantly with plaque age (measured by the nitrogen index LP). Statistical test: Pearson correlation, two tailed. *y-axis has been inverted to account for the difference in labelling design between 10-month- (Experimental design 1) and 18-month-old mice (Experimental design 2), and enable direct comparison of the directionality of gene expression changes with plaque age. Parts of the figure created in BioRender. Szadziewska, A. (2025) https://BioRender.com/4qpojxz

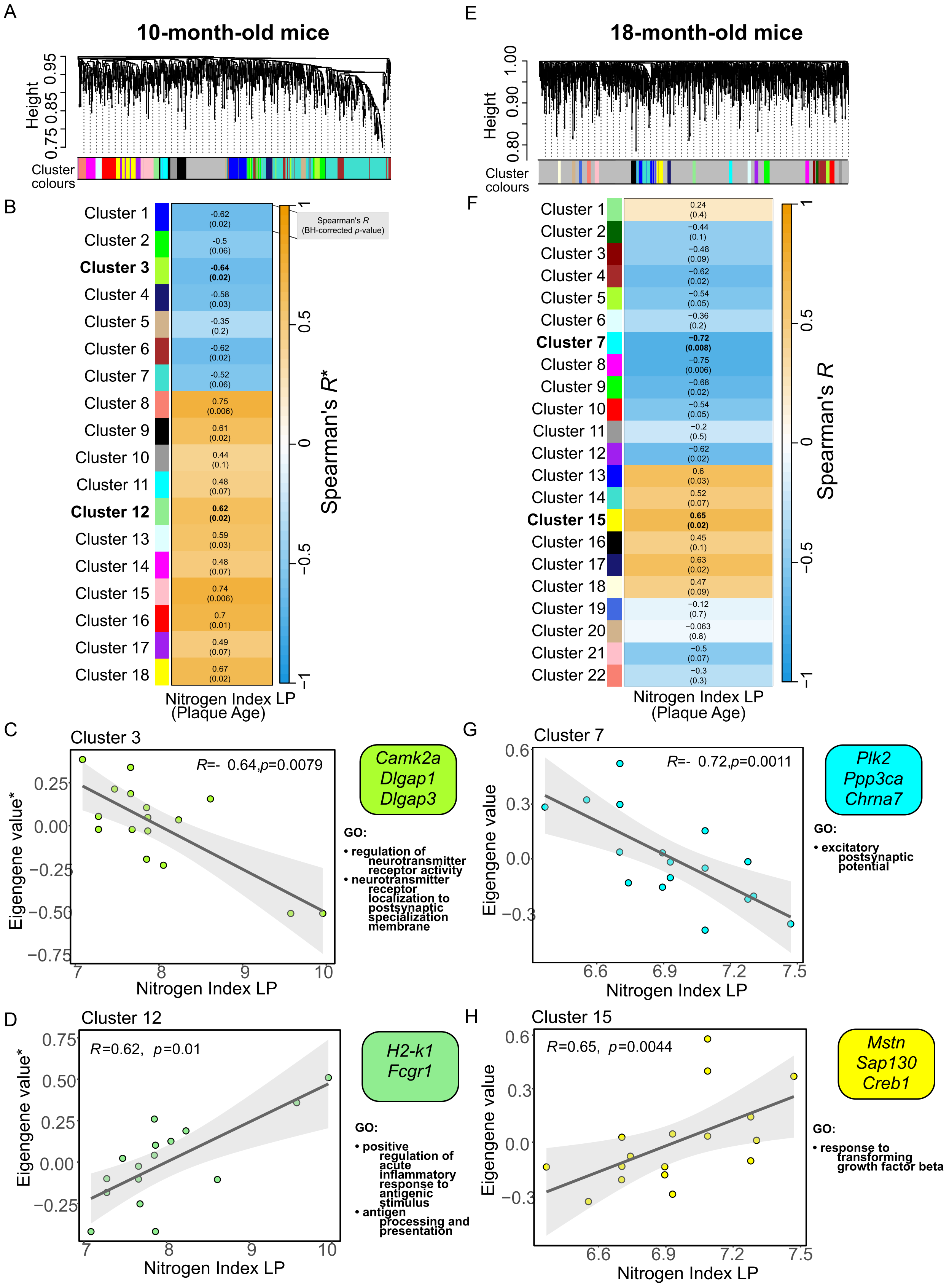

Supplementary Figure 8.

WGCNA analysis of genes significantly correlating with plaque age in 10- (A-D) and 18-month-old mice (E-H). 10-month-old mice.

(A) Cluster dendrogram of genes with respective cluster colours in 10-month-old mice. (B) Heatmap showing the Spearman correlation between each module's Eigengene and the nitrogen index (used as a proxy for plaque age). Clusters of interest are highlighted in bold and further analyzed below. All p-values were corrected for multiple testing using the Benjamini–Hochberg (BH) method. (C) Spearman correlation scatter plot for Cluster 3 (greenyellow cluster), depicting the relationship between plaque age (Nitrogen index LP) and the Eigengene value of Cluster 3 (uncorrected p-value depicted). Proteins of interest and enriched Gene Ontology terms associated with Cluster 3 are listed on the right. (D) Spearman correlation scatter plot for Cluster 12 (lightgreen cluster), depicting the relationship between plaque age (Nitrogen index LP) and the Eigengene value of Cluster 12. *Owing to the different labeling used in 10-month-old mice, the direction of the correlation has been inverted to enable direct comparison with the correlations observed in 18-month-old animals. 18-month-old mice: (E) Cluster dendrogram of genes with respective cluster colours in 18-month-old mice. (F) Heatmap showing the Spearman correlation between each module's Eigengene and the nitrogen index (used as a proxy for plaque age). Clusters of interest are highlighted in bold and further analyzed below. All p-values were corrected for multiple testing using the BH method. (G) Spearman correlation scatter plot for Cluster 7 (cyan cluster), depicting the relationship between plaque age (Nitrogen index LP) and the Eigengene value of Cluster 7. (H) Spearman correlation scatter plot for Cluster 15 (yellow cluster), depicting the relationship between plaque age (Nitrogen index LP) and the Eigengene value of Cluster 15.

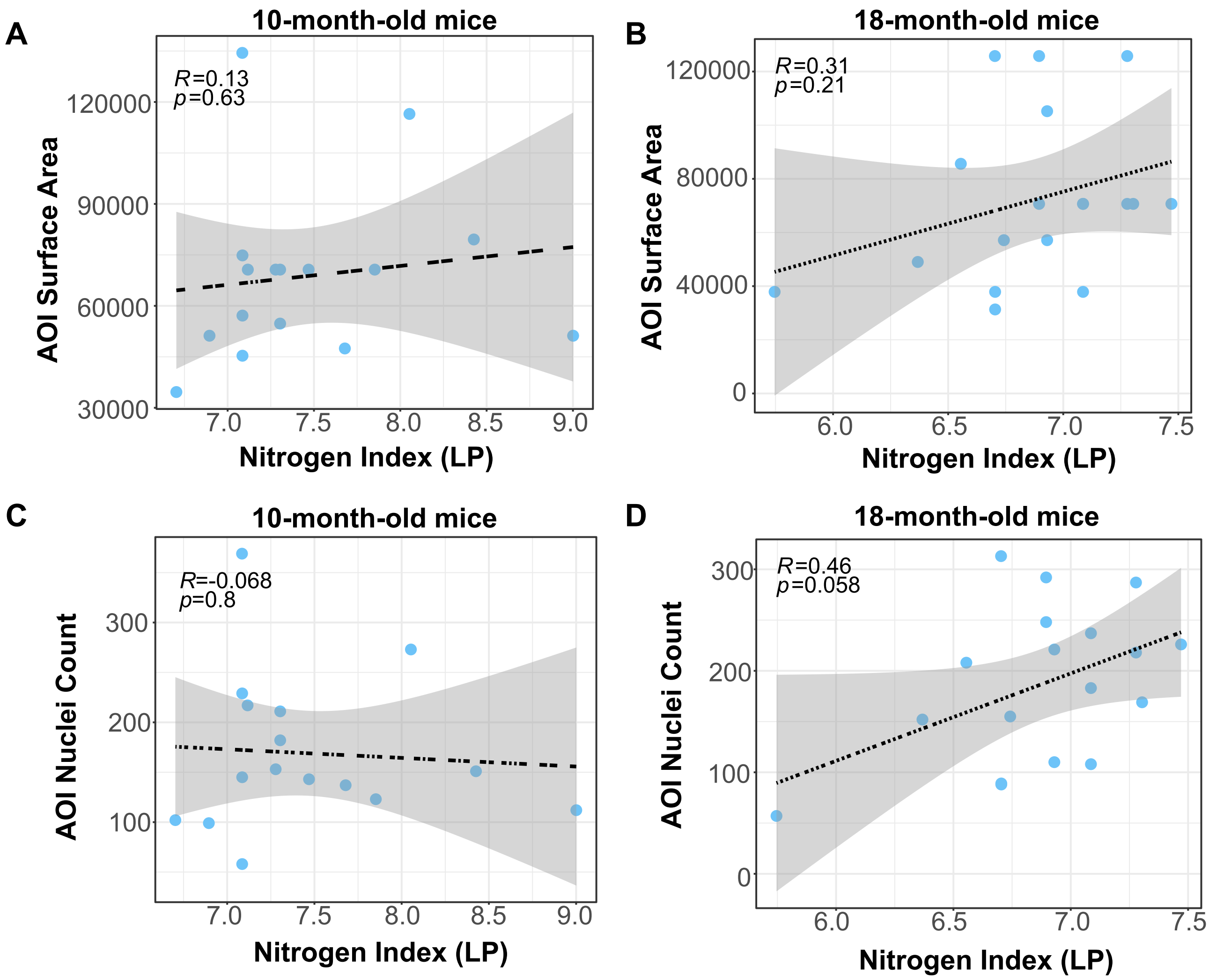

Supplementary Figure 9.

Plaque age, as determined by MALDI MSI, does not correlate with the GeoMx AOI surface area or AOI nuclei count.

Correlation analyses (Pearson, two tailed) of AOI surface area with the corresponding nitrogen index in LP mode in 10-month (**A**) and 18-month-old mice (**B**) revealed no significant association of AOI area with plaque age. Similarly, correlation analyses of AOI nuclei count with plaque age (nitrogen index LP) in 10-month (**C**) and 18-month-old mice (**D**) showed no significant correlation at a significance level of α=0.05.

AOI, Area of Illumination; LP, Linear Positive; MALDI MSI, matrix-assisted laser desorption ionization mass spectrometry imaging.

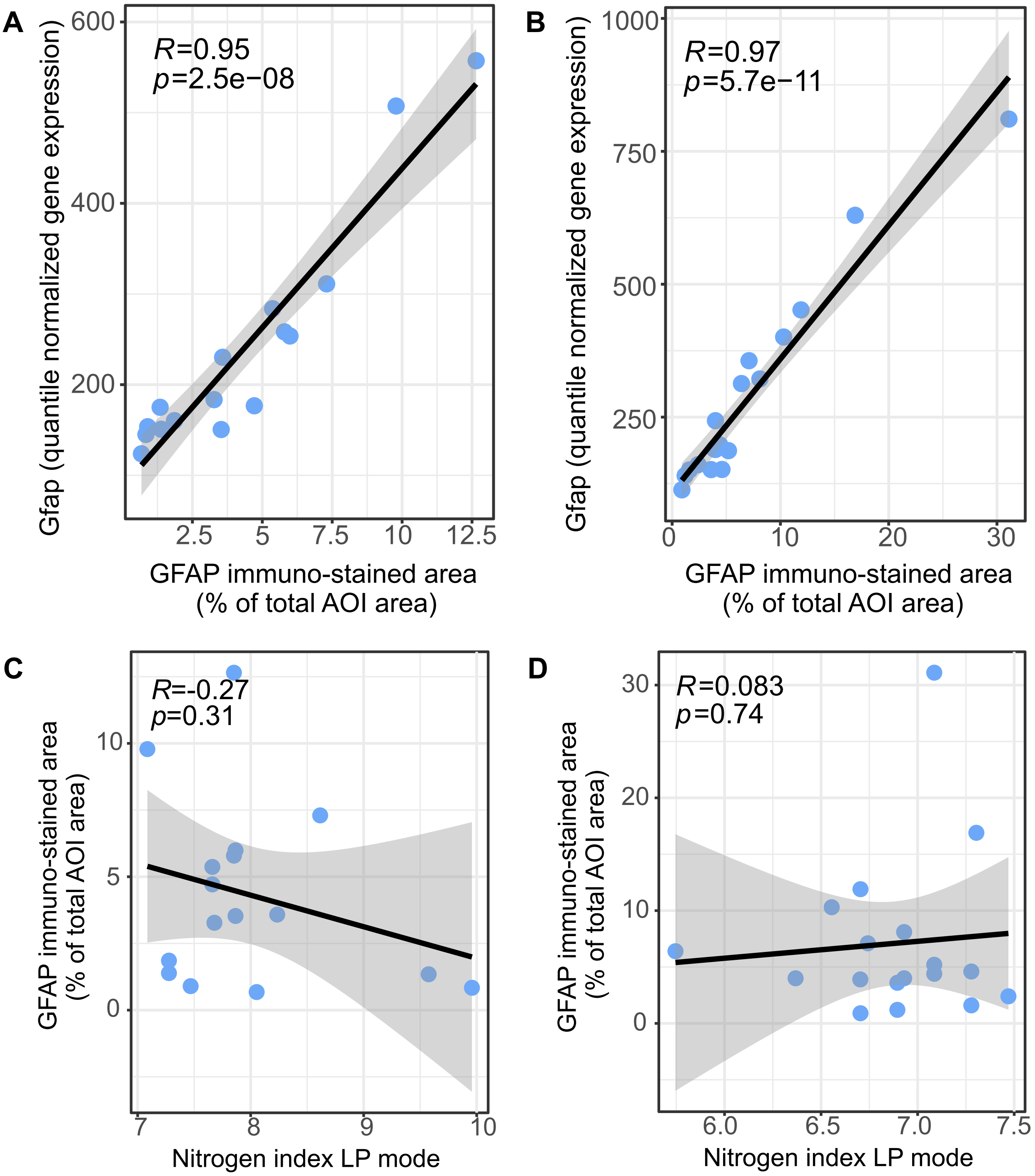

Supplementary Figure 10.

Correlation between GFAP immunostaining, Gfap gene expression, and plaque age.

(**A–B**) Scatter plots showing strong positive correlations between GFAP, astrocyte marker, immuno-stained area (as a percentage of total AOI area) and *Gfap* gene expression in corresponding areas of illumination (AOIs). (**C–D**) Scatter plots showing no significant correlation between plaque age, estimated via nitrogen index measured by MALDI MSI, and GFAP immuno-stained area. Statistical test: Pearson correlation, two tailed.

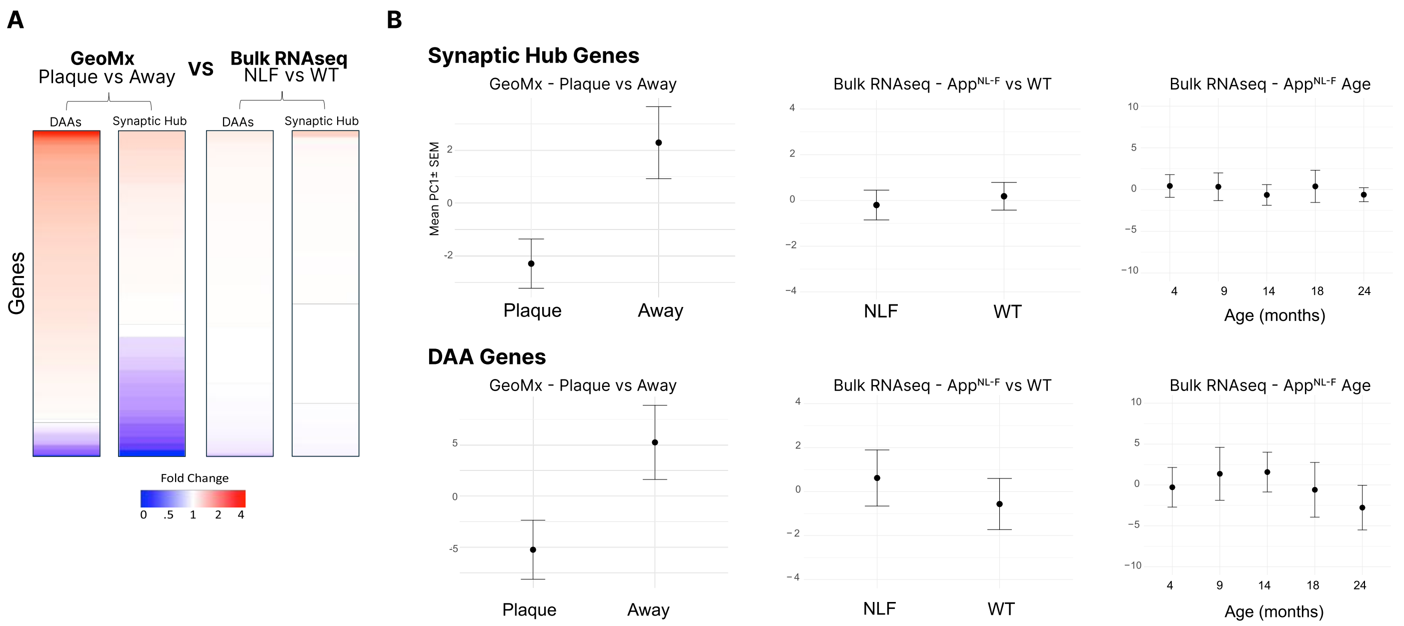

Supplementary Figure 11.

Amyloid induced changes in gene expression detected using either GeoMx spatial transcriptomics or Bulk RNAseq.

(**A**) Heatmaps comparing fold change of Disease Associated Astrocytic genes (DAAs, Habib et al., 2020) and synaptic hub genes (Williams et al., 2021) by use of GeoMx spatial transcriptomic technology using an astrocytic collection (Wood et al., 2022) in plaque vs non-plaque associated areas (n=6) or by use of bulk hippocampal RNA sequencing in 18-month-old wild-type (n=11) vs AppNL-F (n=9) mice. (**B**) Principal Component Analysis of the DAAs and synaptic hub genes performed using data from both GeoMx spatial transcriptomics and Bulk hippocampal RNA sequencing. The first principal component, as a composite score and representative abundance value, was calculated for both synaptic hub and DAA genes (y-axis), and plotted across different regions and conditions (x-axis).

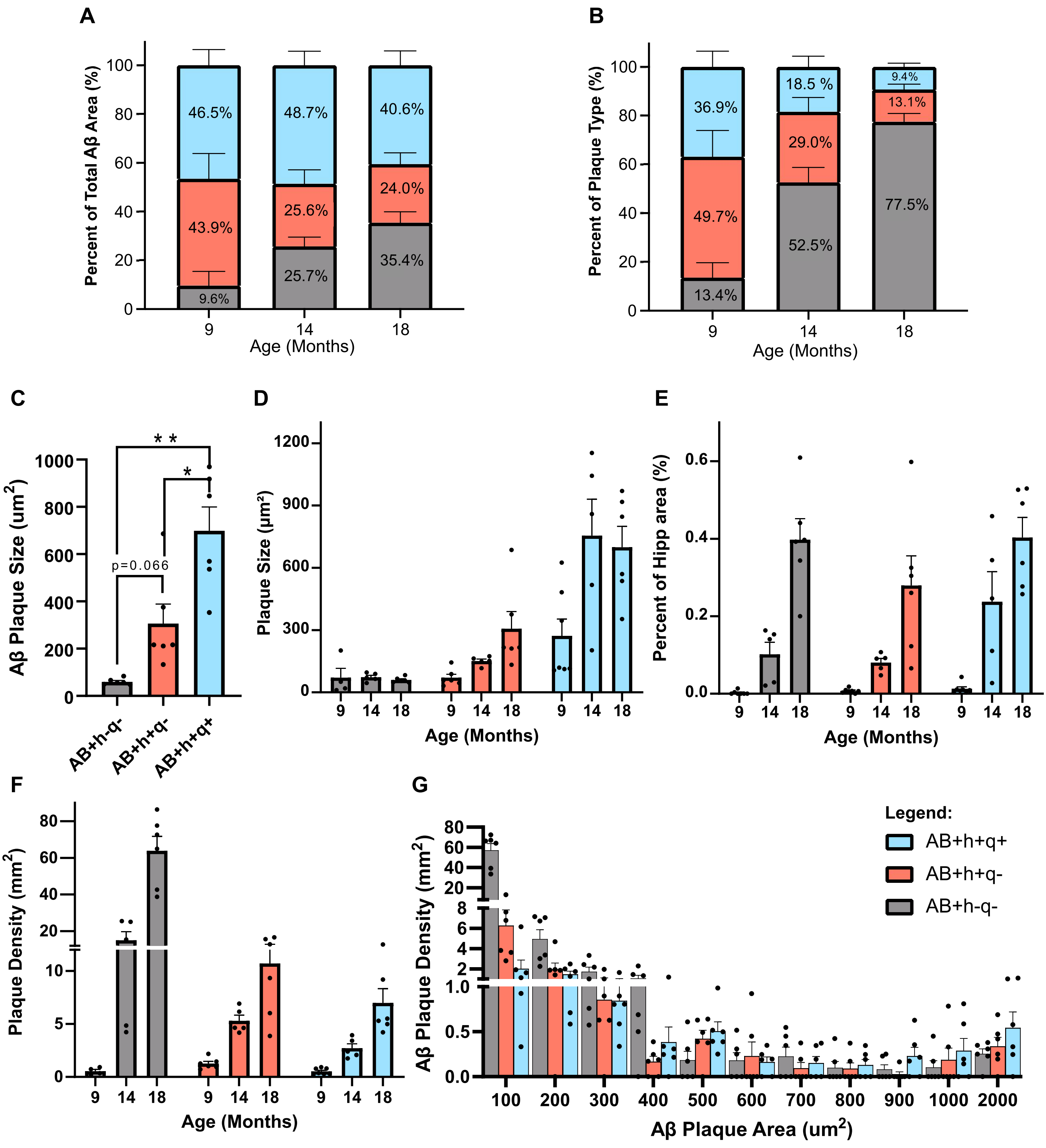

Supplementary Figure 12.

Plaque type characterization in 9-, 14-, and 18-month-old $App^{NL\text{-}F}$ mice

(**A**) Distribution of plaque types as a percentage of the total Aβ positive area over age. (**B**) Distribution of plaque types as a percentage of the total number of Aβ plaques over age. (**C**) Average area covered by individual plaques at 18 months of age. One way ANOVA showed significant differences between AB+h+q+ vs. AB+h+q- (p = 0.0284) and AB+h+q+ vs. AB+h-q- (p = 0.0029). (**D**) Average Aβ plaque size of each plaque type over age. (**E**) Percentage of the hippocampal area occupied by each plaque type. (**F**) Aβ plaque density for each plaque type over age. (**G**) Histogram of Aβ plaque density over plaque size for each plaque type at 18 months of age. Analyses performed on projected z-stacked confocal images. $App^{NL\text{-}F}$ 9m n=7 animals, $App^{NL\text{-}F}$ 14m n=5 animals, $App^{NL\text{-}F}$ 18m n= 6 animals. Data plotted as mean + SEM. *P<0.05, **P<0.01.

Supplementary Table 1.

Relevant MALDI MSI spectral (green) as well as hyperspectral imaging (blue) data for plaques included in the MALDI MSI hyperspectral imaging correlative analysis in 18-month-old App^{NL-F} mice.

Mouse ID	ROI ID	Brain Area (h=hippocampal. c=cortical)	Hyperspectral ratio	Nitrogen Index RP mode
6	ROI1	h	0.0414	1.042
6	ROI2	h	0.0154	0.768
6	ROI3	h	0.1064	0.9
6	ROI4	c	0.1283	1.052
6	ROI5	c	0.3043	1.174
6	ROI6	c	0.3557	1.138
6	ROI7	c	0.1882	1.1
4	ROI8	h	0.1047	0.976
4	ROI9	h	0.0739	0.991
4	ROI10	c	0.6791	1.064
4	ROI11	c	0.1881	0.981
4	ROI12	c	0.2481	1.009
4	ROI13	h	0.1483	1.004
5	ROI14	h	0.1065	0.936
5	ROI15	h	0.1829	0.981
5	ROI16	c	0.3643	1.096
5	ROI17	c	0.4597	1.103
5	ROI18	c	0.3927	1.14
5	ROI19	c	0.3399	1.091
5	ROI20	h	0.3566	1.085
5	ROI21	h	0.3718	1.056

Supplementary Table 2.

Summary statistics of relevant MALDI MSI spectral (green) as well as hyperspectral imaging (blue) data for App^{NL-F} mice (18-month) included in the MALDI MSI hyperspectral imaging correlative analysis.

Mouse ID	Number of plaques	Hyperspectral ratio median (IQR)	Nitrogen Index RP mode median (IQR)
4	6	0.1682 (0.1156 - 0.2331)	0.9975 (0.9835 - 1.0078)
5	8	0.36045 (0.3007 - 0.3770)	1.088 (1.0373 - 1.0978)
6	7	0.1283 (0.0739 - 0.2463)	1.052 (0.9710 - 1.1190)

Supplementary Table 3.

Relevant MALDI MSI spectral (green) as well as spatial transcriptomics (GeoMx, blue) data for plaques included in the MALDI MSI-transcriptomics correlative analysis in 10-month-old App^{NL-F} mice. Peak FWHM corresponds to the nitrogen index in LP mode.

N	Mouse ID	Area of illumination (AOI)	Peak Full Width at Half Maximum (FWHM)	Area of Illumination (AOI) Nuclei Count	Aligned Reads	Surface Area [µm²]	GFAP immuno-stained area [% of AOI area]
1	1	AOI1	7.679	217	42643138	70666.79	3
2	1	AOI2	7.866	211	35445830	70666.79	6
3	1	AOI3	8.053	137	22147367	47461.58	1
4	1	AOI4	8.615	273	65901754	116478.9	7
5	1	AOI5	7.866	182	26149334	54781.43	4
6	2	AOI6	7.277	369	79141433	134451.4	1
7	2	AOI7	9.575	112	17345660	51208.71	1
8	2	AOI8	9.958	151	22644118	79537.16	1
9	2	AOI9	8.235	123	31792631	70666.79	4
10	3	AOI10	7.661	153	44784197	70666.79	5
11	3	AOI11	7.086	102	19665326	34633.56	10
12	3	AOI12	7.852	143	29048319	70666.79	6
13	3	AOI13	7.661	145	24160110	57138.86	5
14	3	AOI14	7.469	99	12738449	51208.71	1
15	3	AOI15	7.852	58	11085605	45323.84	13
16	3	AOI16	7.277	229	31056818	74848.57	2

Supplementary Table 4.

Relevant MALDI MSI spectral (green) as well as spatial transcriptomics (GeoMx, blue) data for plaques included in the MALDI MSI-transcriptomics correlative analysis in 18-month-old App^{NL-F} mice. Peak FWHM corresponds to the nitrogen index in LP mode.

N	Mouse ID	Area of illumination (AOI)	Peak Full Width at Half Maximum (FWHM)	Area of Illumination (AOI) Nuclei Count	Aligned Reads	Surface Area [µm²]	GFAP immuno -stained area [% of AOI area]
1	4	AOI17	6.895	292	37467291	125774.8	1.2
2	4	AOI18	7.278	287	57965148	125774.8	1.6
3	4	AOI19	6.703	313	25269317	125774.8	3.9
4	4	AOI20	7.086	183	18446892	70666.79	31.1
5	4	AOI21	7.47	226	45326376	70666.79	2.4
6	5	AOI22	6.742	155	21195109	57138.86	7.1
7	5	AOI23	7.304	169	18366426	70666.79	16.9
8	5	AOI24	6.93	110	30718229	57138.86	8.1
9	5	AOI25	6.368	152	28736336	49015.89	4
10	5	AOI26	6.93	221	22504633	105212.5	4
11	5	AOI27	6.555	208	16018455	85607.54	10.3
12	6	AOI28	7.086	108	19967819	37872.3	4.4
13	6	AOI29	6.704	89	23299483	31315.05	0.9
14	6	AOI30	5.746	57	12309416	37872.3	6.4
15	7	AOI31	7.086	237	48620337	70666.79	5.2
16	7	AOI32	6.895	248	26616873	70666.79	3.6
17	7	AOI33	7.278	218	21554615	70666.79	4.6
18	7	AOI34	6.704	88	10031020	37872.3	11.9

Supplementary Table 5.

Summary statistics of relevant MALDI MSI spectral (green) as well as hyperspectral imaging (blue) data for App^{NL-F} mice (18-month) included in the MALDI MSI hyperspectral imaging correlative analysis.

Mouse ID	Age [months]	Number of plaques	Peak FWHM median (IQR)	AOI Nuclei Count median (IQR)	Aligned Read median (IQR)	Surface Area median (IQR) [µm2]	GFAP immuno-stained area, median (IQR) [% of AOI area]
1	10	5	7.866 (7.866 - 8.053)	211 (182 - 217)	35445830 (26149334-42643138)	70666.79 (54781.43 - 70666.79)	4 (3-6)
2	10	4	8.905 (7.996-9.671)	137 (120 - 206)	27218375 (21319504 - 43629832)	75101.98 (65802.27 - 93265.72)	1 (1 - 1.8)
3	10	7	7.661 (7.373 - 7.757)	143 (101 - 149)	24160110 (16201888 - 30052569)	57138.86 (48266.28 - 70666.79)	5 (3.5-8)
4	18	5	7.086 (6.895-7.278)	287 (226 - 292)	37467291 (25269317 - 45326376)	125774.8 (70666.79 - 125774.8)	2.4 (1.6-3.9)
5	18	6	6.836 (6.602-6.930)	162 (153 - 198)	21849871 (19073597 - 27178410)	63902.83 (57138.86 - 81872.35)	7.6 (4.8-9.8)
6	18	3	6.704	89	19967819	37872.3	4.4
7	18	4	6.991 (6.847-7.134)	228 (186 - 240)	24085744 (18673716 - 32117739)	70666.79 (62468.16 - 70666.79)	4.9 (4.4-6.9)
