# Supplementary material for: Isotope-encoded spatial biology identifies plaque-age-dependent maturation and synaptic loss in an Alzheimer’s disease mouse model
Source: Nat Commun. 2025 Sep 1;16:8170. doi: 10.1038/s41467-025-63328-y (PMC12402145; doi:10.1038/s41467-025-63328-y)
Supplement: Supplementary file 2 — Reporting Summary [file 41467_2025_63328_MOESM2_ESM.pdf]

nature portfolio

Corresponding author(s): Jörg Hanrieder

Last updated by author(s): Aug 1, 2025

Reporting Summary

Nature Portfolio wishes to improve the reproducibility of the work that we publish. This form provides structure for consistency and transparency in reporting. For further information on Nature Portfolio policies, see our Editorial Policies and the Editorial Policy Checklist.

Statistics

For all statistical analyses, confirm that the following items are present in the figure legend, table legend, main text, or Methods section.

n/a	Confirmed	
☐	☒	The exact sample size (*n*) for each experimental group/condition, given as a discrete number and unit of measurement
☐	☒	A statement on whether measurements were taken from distinct samples or whether the same sample was measured repeatedly
☐	☒	The statistical test(s) used AND whether they are one- or two-sided<br>*Only common tests should be described solely by name; describe more complex techniques in the Methods section.*
☐	☒	A description of all covariates tested
☐	☒	A description of any assumptions or corrections, such as tests of normality and adjustment for multiple comparisons
☐	☒	A full description of the statistical parameters including central tendency (e.g. means) or other basic estimates (e.g. regression coefficient) AND variation (e.g. standard deviation) or associated estimates of uncertainty (e.g. confidence intervals)
☐	☒	For null hypothesis testing, the test statistic (e.g. *F*, *t*, *r*) with confidence intervals, effect sizes, degrees of freedom and *P* value noted<br>*Give P values as exact values whenever suitable.*
☒	☐	For Bayesian analysis, information on the choice of priors and Markov chain Monte Carlo settings
☐	☒	For hierarchical and complex designs, identification of the appropriate level for tests and full reporting of outcomes
☐	☒	Estimates of effect sizes (e.g. Cohen's *d*, Pearson's *r*), indicating how they were calculated

Our web collection on statistics for biologists contains articles on many of the points above.

Software and code

Policy information about availability of computer code

Data collection: Correlation analysis with Plaque Age with MALDI/GeoMx available at https://maciejdulewiczgu.shinyapps.io/MALDI_GEOMX_VOLCANO/

Data analysis: Updated:

Experimental group sizes for the iSILK feeding experiment were based on previous work using the AppNL-G-F mouse model (Michno et al., 2021); to account for potential age-related mortality, one additional animal was included in the 18-month group, resulting in n = 4 for 18-month-old and n = 3 for 10-month-old mice. Group size estimation for immunohistochemistry validation was based on an a priori power analysis using expected effect sizes from pilot data: d = 2.5 for LAMP1 and d = 1.5 for HOMER1. Assuming α = 0.05 and power = 0.8, the analysis indicated minimum sample sizes of n = 3 and n = 5 animals, respectively. For the IHC validation, investigators were blinded to experimental group during image acquisition and analysis. Outlier data points (>2 SD from the mean) were excluded. Specific regions of interest (ROIs) were removed from GeoMx analyses if they showed ambiguous morphology (e.g., overlapping plaques) or insufficient MALDI MSI signal.

ROI selection for GeoMx and MALDI MSI was not blinded or randomized, as all plaques spanning two consecutive sections were required for cross-modality alignment. For MALDI MSI analyses of 15N enrichment in the hippocampus and cortex, as well as center versus periphery comparisons, plaques were randomly selected from within each brain region. In contrast, all identifiable plaques within the hippocampus were included for immunohistochemistry validation, and selection was therefore not randomized. To minimize potential bias, all image acquisition and analysis were performed in random order.

Statistical analyses were performed using GraphPad Prism 9 and RStudio. The specific statistical test used is described in each figure legend.

For comparisons involving paired measurements within plaques (e.g., center vs. periphery), linear mixed-effects models were used to account for intra-plaque replication and inter-mouse variability. Where appropriate, two-sided paired or unpaired t-tests and Mann–Whitney U tests were used for group comparisons, and Pearson correlation was used for continuous variable associations. Post hoc analyses using Sidak's correction were applied only when a statistically significant interaction was detected. In all statistical tests, biological replicates were considered to avoid pseudoreplication.

For transcriptomic correlation analyses, genome-wide comparisons across 19,963 genes were conducted using Pearson correlation coefficients. As the application of multiple testing correction at the gene level (e.g., Benjamini–Hochberg) would restrict detection to only near-perfect correlations ($r \approx 0.9$), correlation P-values were instead used to rank genes for Gene Ontology (GO) enrichment analysis, with correction applied at the pathway level ($FDR < 0.05$).

Data Availability:
The GeoMx transcriptomics data generated in this study are available in the Zenodo 10.5281/zenodo.16676234.
All raw and processed MALDI–MSI data from plaque ROIs are available via an interactive Shiny application at https://maciejdulewiczgu.shinyapps.io/MALDI_GEOMX_VOLCANO/ and 10.5281/zenodo.16676234. Under the 'SPECTRA VIEWER' tab, all MALDI spectra from 18-month and 10-month groups can be viewed and downloaded. These data can be reanalyzed using the IsotopeakeR framework (see Code availability). Under the 'VOLCANO PLOT' and 'GO ENRICHMENT TABLES' tabs, statistical comparisons and correlation analyses of MALDI–MSI and GeoMx transcriptomics are available. The LC-MS/MS data generated in this study have been deposited in the PRIDE database under accession code PXD060410 (IP-LC-MS/MS) and MSV000092311 (proteomics of purified amyloid fibrils).

Code Availability:
The complete code for MALDI–MSI data processing, spectral analysis, and spatial integration with GeoMx transcriptomics is available at:
GitHub: https://github.com/MaciejDulewiczGU/MaldiGeoMxSpatialTranscriptomicsIsotopeaker
Shiny app (IsotopeakeR beta): https://maciejdulewiczgu.shinyapps.io/IsotopeakeR_Beta
Instructions for reanalyzing spectra downloaded from the 'SPECTRA VIEWER' tab of the MALDI–GeoMx portal using IsotopeakeR are provided in the GitHub repository.

Software +Versions (V):
R (v4.3.1), used packages below:
preprocessCore
clusterProfiler
GOplot
StringDB
tidyr
msigdbr
WGCNA
shiny
plotly
dplyr
signal
GraphPad Prism 9
ImageJ v2.16
Qupath v0.5.1
Zen Black 2.3
ZEISS ZEN v3.9
GeoMX NGS Pipeline 2.3.4
PEAKS Studio v12.5
DTASelect2 v2.1.3
flexImaging v5.0
ProLuCID (v n/a)
RawConverter (https://github.com/proteomicsyates/RawConverter)
IP2 (http://www.integratedproteomics.com/)

For manuscripts utilizing custom algorithms or software that are central to the research but not yet described in published literature, software must be made available to editors and reviewers. We strongly encourage code deposition in a community repository (e.g. GitHub). See the Nature Portfolio guidelines for submitting code & software for further information.

Data

Policy information about availability of data

All manuscripts must include a data availability statement. This statement should provide the following information, where applicable:

- Accession codes, unique identifiers, or web links for publicly available datasets
- A description of any restrictions on data availability
- For clinical datasets or third party data, please ensure that the statement adheres to our policy

Updated:
The GeoMx transcriptomics data generated in this study are available in the Zenodo: 10.5281/zenodo.16676234.
All raw and processed MALDI–MSI data from plaque ROIs are available via an interactive Shiny application at https://maciejdulewiczgu.shinyapps.io/MALDI_GEOMX_VOLCANO/ and 10.5281/zenodo.16676234. Under the 'SPECTRA VIEWER' tab, all MALDI spectra from 18-month and 10-month groups can be viewed and downloaded. These data can be reanalyzed using the IsotopeakeR framework (see Code availability). Under the 'VOLCANO PLOT' and 'GO ENRICHMENT

TABLES' tabs, statistical comparisons and correlation analyses of MALDI–MSI and GeoMx transcriptomics are available. The LC-MS/MS data generated in this study have been deposited in the PRIDE database under accession code PXD060410 (IP-LC-MS/MS) and MSV000092311 (proteomics of purified amyloid fibrils).

Research involving human participants, their data, or biological material

Policy information about studies with human participants or human data. See also policy information about sex, gender (identity/presentation), and sexual orientation and race, ethnicity and racism.

Reporting on sex and gender	*Use the terms sex (biological attribute) and gender (shaped by social and cultural circumstances) carefully in order to avoid confusing both terms. Indicate if findings apply to only one sex or gender; describe whether sex and gender were considered in study design; whether sex and/or gender was determined based on self-reporting or assigned and methods used. Provide in the source data disaggregated sex and gender data, where this information has been collected, and if consent has been obtained for sharing of individual-level data; provide overall numbers in this Reporting Summary. Please state if this information has not been collected. Report sex- and gender-based analyses where performed, justify reasons for lack of sex- and gender-based analysis.*
Reporting on race, ethnicity, or other socially relevant groupings	*Please specify the socially constructed or socially relevant categorization variable(s) used in your manuscript and explain why they were used. Please note that such variables should not be used as proxies for other socially constructed/relevant variables (for example, race or ethnicity should not be used as a proxy for socioeconomic status). Provide clear definitions of the relevant terms used, how they were provided (by the participants/respondents, the researchers, or third parties), and the method(s) used to classify people into the different categories (e.g. self-report, census or administrative data, social media data, etc.) Please provide details about how you controlled for confounding variables in your analyses.*
Population characteristics	*Describe the covariate-relevant population characteristics of the human research participants (e.g. age, genotypic information, past and current diagnosis and treatment categories). If you filled out the behavioural & social sciences study design questions and have nothing to add here, write "See above."*
Recruitment	*Describe how participants were recruited. Outline any potential self-selection bias or other biases that may be present and how these are likely to impact results.*
Ethics oversight	*Identify the organization(s) that approved the study protocol.*

Note that full information on the approval of the study protocol must also be provided in the manuscript.

Field-specific reporting

Please select the one below that is the best fit for your research. If you are not sure, read the appropriate sections before making your selection.

☒ Life sciences ☐ Behavioural & social sciences ☐ Ecological, evolutionary & environmental sciences

For a reference copy of the document with all sections, see nature.com/documents/nr-reporting-summary-flat.pdf

Life sciences study design

All studies must disclose on these points even when the disclosure is negative.

Sample size	Sample size varied throughout study, calculated for each experiment using power analysis, G*Power.
Data exclusions	GeoMx: Probes were excluded from analysis if counts were too low or failed the Grubbs outlier test according to NanoString guidelines. MALDI: Six ROIs in the 10-month-old group and three ROIs in the 18-monthold group were excluded from further analysis due to ambiguous spot morphology >1 plaque or very low intensity in MALDI results
Replication	GeoMx; n=4 biological/N=6 plaques per animal (sum N=24);<br>SILK and MALDI MSI: n=3 and n=4 biol. replicates for 10mo and 18mo mice; N=6 plaques per animal<br>IHC: LCO/HOMER: n=6 biol. replicates; LCO/LAMP1: n=7 biol. replicates
Randomization	For IHC analysis, all samples were randomized
Blinding	the researches acquiring the MALDI MSI, IHC and GeoMx data were blinded

Reporting for specific materials, systems and methods

We require information from authors about some types of materials, experimental systems and methods used in many studies. Here, indicate whether each material, system or method listed is relevant to your study. If you are not sure if a list item applies to your research, read the appropriate section before selecting a response.

Materials & experimental systems

n/a	Involved in the study
	[x] Antibodies
[x]	Eukaryotic cell lines
[x]	Palaeontology and archaeology
	[x] Animals and other organisms
[x]	Clinical data
[x]	Dual use research of concern
[x]	Plants

Methods

n/a	Involved in the study
[x]	ChIP-seq
[x]	Flow cytometry
[x]	MRI-based neuroimaging

Antibodies

Antibodies used

Mouse anti-GFAP Alexa-Fluor 488 conjugate (1:500, Invitrogen, #53-9892-82),
Mouse anti- Aβ40/42 Alexa Fluor 594 conjugate (1:500, Nanostring, #121301306).
Mouse anti-Aβ (1:500, BioLegend, 6E10, #SIG-39320)
Mouse anti-Aβ (BioLegend, 4G8, #SIG-39220)
rabbit anti-Aβ (1:500, Thermo Fisher Scientific, #700254)
chicken anti-HOMER1 (1:200, Synaptic Systems, #160001)
rat anti-LAMP1 (1:500, Abcam, #ab25245))
goat anti-mouse AF594 (1:500, Thermo Fisher Scientific, #A11032)
goat anti-rabbit AF594 (1:500, Thermo Fisher Scientific, #A11037)
goat anti-chicken AF647 (1:500, Thermo Fisher Scientific, #A-21449)
Donkey anti-rat AF594 (1:500, Thermo Fisher Scientific, #A-21209))

Validation

Mouse anti-Aβ (6E10, #SIG-39320)
Link: https://www.biolegend.com/en-gb/products/purified-anti-beta-amyloid-1-16-antibody-11228?GroupID=BLG15648

Datasheet: https://d1spbj2x7qk4bg.cloudfront.net/en-gb/products/purified-anti-beta-amyloid-1-16-antibody-11228?displayInline=true&filename=Purified%20anti-%CE%B2-Amyloid,%201-16%20%20Antibody.pdf&leftRightMargin=15&pdf=true&topBottomMargin=15&v=20241208073714

Validation Description: Western blot of purified anti-β-amyloid, 1-16 antibody (clone 6E10)

Citations (383):
1. Abud EM et al. 2017. Neuron. 94(2):278-293 . PubMed
2. Wang X, et al. 2019. Cell Res. 29:787. PubMed
3. Eede P, et al. 2020. EMBO Rep. 21:e48530. PubMed
4. Sogorb-Esteve A, et al. 2018. Mol Neurobiol. 55:5047. PubMed
5. Turnbull MT, et al. 2018. Front Mol Neurosci. 11:51. PubMed
6. Singh N, et al. 2022. Sci Adv. 8:eabo1286. PubMed
7. Ye Q, et al. 2022. Neurobiol Dis. 172:105820. PubMed
8. Guo T, et al. 2022. J Neurosci. . PubMed
9. Vasilopoulou MA, et al. 2022. Redox Biol. 56:102462. PubMed
10. Yu H, et al. 2023. Alzheimers Dement. 19:2365. PubMed
11. Williams D, et al. 2023. Sci Rep. 13:2337. PubMed
12. Rimal S, et al. 2023. EMBO Rep. 24:e55548. PubMed

Mouse anti-Aβ (BioLegend, 4G8, #SIG-39220)
Link: https://www.biolegend.com/ja-jp/products/purified-anti-beta-amyloid-17-24-antibody-11233
Datasheet: https://www.biolegend.com/ja-jp/products/purified-anti-beta-amyloid-17-24-antibody-11233

Citations:

Jovic M, et al. 2019. PLoS One. 14:e0216726. PubMed
Bishay J, et al. 2022. Sci Rep. 12:15287. PubMed
Libard S, et al. 2022. J Alzheimers Dis. 90:1601. PubMed
Williams D, et al. 2023. Sci Rep. 13:2337. PubMed
Natarajan C, et al. 2023. Int J Mol Sci. 24:. PubMed
Campbell NB, et al. 2023. Int J Mol Sci. 24:. PubMed
Lia A, et al. 2023. Nat Commun. 14:1590. PubMed
Jiang Y, et al. 2022. Nat Aging. 2:616. PubMed
Woelfle S, et al. 2023. BMC Biol. 21:113. PubMed
Illouz T, et al. 2021. Vaccine. 39:4817. PubMed
Schober R, et al. 2021. Neuropathology. 41:366. PubMed
Abrahamson EE, et al. 2022. Brain. 145:2161. PubMed

Rabbit anti-Aβ (#700254)
Link: https://www.thermofisher.com/antibody/product/beta-Amyloid-Antibody-clone-H31L21-Recombinant-Monoclonal/700254

Data sheet: https://www.thermofisher.com/order/genome-database/dataSheetPdf?productype=antibody&productsubtype=antibody_primary&productId=700254&version=Local

Validation Description:
Cross reactivity to Abeta [1-40] is not observed in sandwich ELISA. In addition, in antigen ELISA cross-reactivity is not observed with Abeta [1-37], Abeta [1-38], Abeta [1-40], or Abeta [1-43] when used at low antibody concentrations (up to 30 ng/mL).

Recombinant rabbit monoclonal antibodies are produced using in vitro expression systems. The expression systems are developed by cloning in the specific antibody DNA sequences from immunoreactive rabbits. Then, individual clones are screened to select the best candidates for production. The advantages of using recombinant rabbit monoclonal antibodies include: better specificity and sensitivity, lot-to-lot consistency, animal origin-free formulations, and broader immunoreactivity to diverse targets due to larger rabbit immune repertoire.

Immunohistochemistry analysis of beta-Amyloid in formalin-fixed, paraffin embedded human brain (left) and transgenic mouse tissue that expresses FAD mutant APP and PS1 (right) using a beta-Amyloid monoclonal antibody (Product # 700254) at a dilution of 1 µg/mL. Results show strong cytoplasmic staining in amyloid plaque.

Citations (36 total):
Immunohistochemistry:
1. Weible AP,Wehr M: Amyloid Pathology in the Central Auditory Pathway of 5XFAD Mice Appears First in Auditory Cortex. 2022
2. Jankovska N,Olejar T,Matej R: Extracellular Protein Aggregates Colocalization and Neuronal Dystrophy in Comorbid Alzheimer's and Creutzfeldt-Jakob Disease: A Micromorphological Pilot Study on 20 Brains. 2021
Westerns
1. Authors: Zhang T,Shen Y,Guo Y,Yao J: Identification of key transcriptome biomarkers based on a vital gene module associated with pathological changes in Alzheimer's disease. 2021

Chicken anti-HOMER1 (#160006)
Link: https://sysy.com/product/160006

Data sheet: https://sysy.com/product-factsheet/SySy_160006

Validation Description:
Specific for Homer 1. According to Soloviev et al. (2000), aa 1 - 180 are present in isoforms a, b, c and d.
Western blot against Homer1, with associated molecular weight.

Citations:
Inhibition of LRRK2 kinase activity promotes anterograde axonal transport and presynaptic targeting of α-synuclein. Brzozowski CF, Hijaz BA, Singh V, Gcwensa NZ, Kelly K, Boyden ES, West AB, Sarkar D, Volpicelli-Daley LA Acta neuropathologica communications (2021) 91: 180. . ICC, IHC, EXM; tested species: mouse

A genetic variant of the Wnt receptor LRP6 accelerates synapse degeneration during aging and in Alzheimer's disease. Jones ME, Büchler J, Dufor T, Palomer E, Teo S, Martin-Flores N, Boroviak K, Metzakopian E, Gibb A, Salinas PC Science advances (2023) 92: eabo7421. . ICC, IHC; tested species: mouse

Cationic peptides erase memories by removing synaptic AMPA receptors through endophilin-mediated endocytosis. Beier K, Stokes E, Zhuang Y, Toledano M, Vasquez J, Azouz G, Hui M, Tyler I, Shi X, Aoto J Research square (2023) : . . ICC, EXM; tested species: mouse

Interleukin-4 receptor signaling modulates neuronal network activity. Hanuscheck N, Thalman C, Domingues M, Schmaul S, Muthuraman M, Hetsch F, Ecker M, Endle H, Oshaghi M, Martino G, Kuhlmann T, et al. The Journal of experimental medicine (2022) 2196: . . ICC, IHC; tested species: human,mouse

Rat anti-LAMP1 (#ab25245))
Link: https://www.abcam.com/en-us/products/primary-antibodies/lamp1-antibody-1d4b-ab25245?srsltid=AfmBOopIGK9wsR75jFmwurTjD0XYfhMaW7irFtMhonMVqToJNk4L5wdm

Data sheet: https://doc.abcam.com/datasheets/active/ab25245/en-us/lamp1-antibody-1d4b-ab25245.pdf

Validation Description:
Abcam – 'Tested We have tested this species and application combination and it works. It is covered by our product promise.'
Flow cytometry, Immunohistochemistry validatory experiments

Citations (211):
IHC:
1. Yuka Mimura-Yamamoto et. al. Dynamics and function of CXCR4 in formation of the granule cell layer during hippocampal

development. 2017
2. Ciaran E Finn et. al. A second wave of Salmonella T3SS1 activity prolongs the lifespan of infected epithelial cells. 2017
3. Fabio Catalano et al. Tagged IDS causes efficient and engraftment-independent prevention of brain pathology during lentiviral gene therapy for Mucopolysaccharidosis type II. 2023

Alexa Fluorophores:
'With more than 80,000 publications, our Alexa Fluor secondary antibodies are reliable research tools. Browse our comprehensive portfolio of Alexa Fluor and Alexa Fluor Plus secondary antibodies for fluorescent detection of primary antibodies in a wide range of applications, such as cell imaging, flow cytometry, and western blotting.'

Secondary antibodies are affinity-purified antibodies with well-characterized specificity for mouse immunoglobulins and are useful in the detection, sorting or purification of its specified target.

Goat anti-mouse AF594 (#A11032)
Link: https://www.thermofisher.com/antibody/product/Goat-anti-Mouse-IgG-H-L-Highly-Cross-Adsorbed-Secondary-Antibody-Polyclonal/A-11032

Goat anti-rabbit AF594 (#A11037)
Link: https://www.thermofisher.com/antibody/product/Goat-anti-Rabbit-IgG-H-L-Highly-Cross-Adsorbed-Secondary-Antibody-Polyclonal/A-11037

Goat anti-chicken AF647 (#A-21449)
Link: https://www.thermofisher.com/antibody/product/Goat-anti-Chicken-IgY-H-L-Secondary-Antibody-Polyclonal/A-21449

Donkey anti-rat AF594 (#A-21209))
Link: https://www.thermofisher.com/antibody/product/Donkey-anti-Rat-IgG-H-L-Highly-Cross-Adsorbed-Secondary-Antibody-Polyclonal/A-21209

Mouse anti-GFAP Alexa-Fluor 488 conjugate (Invitrogen, #53-9892-82),

Validation description:
Applications Tested: This GA5 antibody has been tested by immunocytochemistry of fixed and permeabilized C6 cells and by immunohistochemistry of formalin-fixed paraffin embedded human tissue using low pH antigen retrieval.

This GA5 antibody has been reported for use in intracellular staining followed by flow cytometric analysis, immunohistology staining of frozen and FFPE tissue sections, and immunocytochemistry.

Link: https://www.thermofisher.com/antibody/product/GFAP-Antibody-clone-GA5-Monoclonal/53-9892-82

Data Sheet: https://www.thermofisher.com/order/genome-database/dataSheetPdf?producttype=antibody&productsubtype=antibody_primary&productId=53-9892-82&version=Local

Citations (51):
1. Le Roy L,Amara A,Le Roux C,Bocher O,Létondor A,Benz N,Timsit S: Principal component analysis, a useful tool to study cyclin-dependent kinase-inhibitor's effect on cerebral ischaemia. 2024
2. Zhou XY,Lin B,Chen W,Cao RQ,Guo Y,Said A,Khan T,Zhang HL,Zhu YM. The brain protection of MLKL inhibitor necrosulfonamide against focal ischemia/reperfusion injury associating with blocking the nucleus and nuclear envelope translocation of MLKL and RIP3K. 2023

Mouse anti- Aβ40/42 Alexa Fluor 594 conjugate (Nanostring, #121301306).
Proprietary antibody used by Nanostring. Validated on fixed and frozen, human and mouse tissue.
https://nanostring.com/resources/morphology-marker-clone-list/

Animals and other research organisms

Policy information about studies involving animals; ARRIVE guidelines recommended for reporting animal research, and Sex and Gender in Research

Laboratory animals	Male and Female APP knock-in mice (APPNL-F,) developed and gifted by the Saido group at the RIKEN Centre for Brain Science, Japan) carrying humanized Aβ sequence, along with the Swedish mutation (KM670/671NL) on exon 16 and the Beyreuther/Iberian mutations (I716F) on exon 17 were used in the study. See - Saito, T., Matsuba, Y., Mihira, N. et al. Single App knock-in mouse models of Alzheimer's disease. Nat Neurosci 17, 661–663 (2014). https://doi.org/10.1038/nn.3697
Wild animals	*Provide details on animals observed in or captured in the field; report species and age where possible. Describe how animals were caught and transported and what happened to captive animals after the study (if killed, explain why and describe method; if released, say where and when) OR state that the study did not involve wild animals.*
Reporting on sex	SILK/MALDI/GeoMx: Mixed sex mice throughout the study. Sex not considered in current study due to low sample size.

	For immunohistochemistry studies , mice were divided equally into Male and Female. The sex split for every experiment is provided in the figure legend.
Field-collected samples	*For laboratory work with field-collected samples, describe all relevant parameters such as housing, maintenance, temperature, photoperiod and end-of-experiment protocol OR state that the study did not involve samples collected from the field.*
Ethics oversight	All procedures and experiments on mice were performed at UCL with local ethical approval (06/05/2016) and in agreement with guidelines of the Institutional Animal Care and Use Committee (IACUC) and the Animals (Scientific Procedures) Act 1986.

Note that full information on the approval of the study protocol must also be provided in the manuscript.

Plants

Seed stocks	*Report on the source of all seed stocks or other plant material used. If applicable, state the seed stock centre and catalogue number. If plant specimens were collected from the field, describe the collection location, date and sampling procedures.*
Novel plant genotypes	*Describe the methods by which all novel plant genotypes were produced. This includes those generated by transgenic approaches, gene editing, chemical/radiation-based mutagenesis and hybridization. For transgenic lines, describe the transformation method, the number of independent lines analyzed and the generation upon which experiments were performed. For gene-edited lines, describe the editor used, the endogenous sequence targeted for editing, the targeting guide RNA sequence (if applicable) and how the editor was applied.*
Authentication	*Describe any authentication procedures for each seed stock used or novel genotype generated. Describe any experiments used to assess the effect of a mutation and, where applicable, how potential secondary effects (e.g. second site T-DNA insertions, mosiacism, off-target gene editing) were examined.*
